# Publication activity in medical education research: A descriptive analysis of submissions to the GMS Zeitschrift für Medizinische Ausbildung in 2007-2015

**DOI:** 10.3205/zma001109

**Published:** 2017-08-15

**Authors:** Jan Matthes, Marianne Giesler, Michaela Wagner-Menghin, Monika Himmelbauer, Ingrid Preusche, Katrin Schüttpelz-Brauns

**Affiliations:** 1 University of Cologne, Institute II, Center for Pharmacology, Cologne, Germany; 2 University of Freiburg, Medical Faculty, Office of Student Affairs, Centre for Evaluation of Teaching in Medicine Baden-Württemberg, Freiburg, Germany; 3 Medical University of Vienna, Teaching Center, Vienna, Austria; 4 University of Veterinary Medicine, Teaching Center, Vienna, Austria; 5 University Medicine Mannheim, Medical Faculty Mannheim at Heidelberg University, Department of Undergraduate Education and Educational Development, Mannheim, Germany

**Keywords:** medical education, education research, publications, data analysis

## Abstract

**Objectives: **The significance of medical education research has increased internationally. In this context we investigated whether, and if so, how the quantity and quality of scientific papers reviewed and/or published by the *GMS Zeitschrift für Medizinische Ausbildung* (GMS Z Med Ausbild) changed.

**Methods: **The quantity and ratio of original papers, project reports and reviews submitted to or published in the GMS Z Med Ausbild were analysed. Published scientific articles were investigated in regard to the quality features “study type” and “mode of data collection” as well as the background (university affiliation) of the last authors. The citation frequency within the first five years after PubMed listing was compared to the one of BMC Medical Education in the corresponding period.

**Results: **The number of submitted scientific manuscripts increased steadily. Most of the submissions and publications are original papers. For publications explorative studies and prospective data collection are most common. A shift over time is not observed. 16% of the published works come from one and 36% from four of the in total 39 universities represented by the last authors. The development of the citation frequency of articles published in GMS Z Med Ausbild is similar to that of BMC Medical Education.

**Conclusion: **The rising number of submissions indicates an increasing significance of medical education research in German-speaking countries. The development of the number of citations reflects the growing appreciation of GMS Z Med Ausbild also indicated by the increasing number of online accesses. Our findings that study type and mode of data collection did not change has to be interpreted with caution since among other things choice and correct application of adequate methods are crucial regarding a scientific work’s quality, too. These aspects, however, were not investigated in this paper.

## Zusammenfassung

**Zielsetzung: **Der Stellenwert Medizinischer Ausbildungsforschung hat international zugenommen. In diesem Kontext haben wir untersucht, ob und ggf. wie sich die Anzahl und die Qualität der wissenschaftlichen Arbeiten verändert hat, die von der GMS Z Med Ausbild begutachtet bzw. publiziert wurden.

**Methodik: **Es wurden Anzahl und Anteil von 2007-2015 bei der GMS Z Med Ausbild eingereichten bzw. publizierten Originalarbeiten, Projektberichten und Übersichtsarbeiten analysiert. Veröffentlichte wissenschaftliche Beiträge wurden in Bezug auf die Qualitätsmerkmale Studientyp und Art der Datenerhebung sowie Herkunft (Hochschulzugehörigkeit) der Letztautor/inn/en untersucht. Die Zitationshäufigkeit innerhalb der ersten fünf Jahre nach PubMed-Listung wurde mit der von BMC Medical Education im entsprechenden Zeitraum verglichen.

**Ergebnisse:** Die Zahl eingereichter wissenschaftlicher Manuskripte nahm ständig zu. Bei Einreichungen und Publikationen sind Originalarbeiten der häufigste Publikationstyp. Bei Publikationen sind explorative Studien und prospektive Datenerhebungen am häufigsten. Eine Veränderung über die Zeit ist hierbei nicht erkennbar. 16% der publizierten Arbeiten stammen aus einer und 36% aus vier von insgesamt 39 durch die Letztautor/inn/en vertretenen Hochschulen. Die Entwicklung der Zitationshäufigkeit von in der GMS Z Med Ausbild veröffentlichten Artikeln ist mit der von BMC Medical Education vergleichbar.

**Schlussfolgerung:** Die steigende Zahl an Einreichungen spricht für eine Zunahme des Stellenwerts Medizinischer Ausbildungsforschung im deutschsprachigen Raum. Die Entwicklung der Zitationszahlen spiegelt eine zunehmende Wahrnehmung der Zeitschrift wider, wie sie sich auch in der steigenden Zahl von Online-Zugriffen zeigt. Dass sich bei den publizierten Arbeiten keine Veränderung von Studienart oder Art der Datenerhebung zeigt, lässt nur sehr bedingt Rückschlüsse auf die Qualität der Forschung zu, da z.B. die Auswahl angemessener Methoden und deren adäquate Umsetzung von wesentlicher Bedeutung sind. Diese Aspekte wurden in der vorliegenden Arbeit allerdings nicht untersucht.

## 1. Introduction

### 1.1. Background

Medical education research has globally gained in significance over the last decades. In Germany, Austria and Switzerland it has also come to the fore: thus the German Association for Medical Education (GMA), which is part of the Association of the Scientific Medical Societies in Germany (Arbeitsgemeinschaft der Wissenschaftlichen Medizinischen Fachgesellschaften e.V., AWMF) since 1986, recorded steadily increasing numbers of members during the last 15 years [1]. Additionally, a growing publication activity of authors from German-speaking countries in international English-language journals on medical education research can be observed [2]. In a comparison of PubMed listed publications on medical education research from 1974-2014 Germany ranked fifth among the fifteen most frequently represented countries of origin, however, far from the leading countries USA, Great Britain and Canada [3].

The Journal Citation Reports® [https://jcr.incites.thomsonreuters.com] issued by Thomas Reuters list internationally significant and well received journals, currently including journals like Academic Medicine, Medical Education or Medical Teacher, that all are relevant for medical education research. These journals have not only a tradition in common that goes back decades, but also share English as the only language of publication and authors mainly coming from Anglo-American countries [3], [4], [5]. BMC Medical Education is an open access journal published in English, which was first issued in 2001. Germany contributes the freely available GMS Zeitschrift für Medizinische Ausbildung (GMS Z Med Ausbild), which represents the GMA. The journal was first issued in 1984 under the title “Medizinische Ausbildung“ and was published as a supplement to “Das Gesundheitswesen” from 1998 until 2004 [6]. Since 2005 the journal is issued as “open access“ by the publisher German Medical Science (GMS). Formerly known as “GMS Zeitschrift für Medizinische Ausbildung“, the journal’s name is “GMS Journal for Medical Education” (GMS J Med Educ) since 2016. The declared mission of the journal is “to contribute to furthering scientific knowledge in the German-speaking countries as well as internationally and thus to foster the improvement of teaching and learning and to build an evidence base for undergraduate and graduate education” [http://www.egms.de/en/journals/zma/about.htm].

In recent years the publishers focused on the internationalisation of the journal: since 2010 the journal is listed in PubMed [http://www.ncbi.nlm.nih.gov/pubmed] and since 2011 the articles are published both in German and English language [1] while the editorial team gained members from various parts of the world [7]. The publishers also seek for the journal to be included in the Journal Citation Reports® that is combined with the yearly attribution of the journal impact factor as the best-known bibliometric indicator [8].

#### 1.2. Research Question

The significance of medical education research was recently emphasised by a statement from the German Council of Science and Humanities (Wissenschaftsrat) regarding recommendations for further development of medical undergraduate training in Germany [9]. The council recommended that universities and countries should “strengthen medical education research in Germany and network it systematically” [https://www.wissenschaftsrat.de/download/archiv/4017-14_Executive-Summary.pdf]. The GMA now faces the question which further steps should be taken to promote education research in German-speaking countries. In this context it is of great interest whether the increasing significance of and the growing expectations in education research are likewise reflected in German-speaking countries. Since GMS Z Med Ausbild/J Med Educ comprises most German publications on medical education research, we addressed this journal to descriptively answer the following questions:

- How did the number of scientific articles that were submitted to, published in and/or rejected by GMS Z Med Ausbild change over the years?
- Is there a change regarding the type of submission (original paper, project report and review) and the study type (e.g. explorative or experimental) in publications of the GMS Z Med Ausbild?

Additionally it would be interesting to find out whether the activities of the GMS Z Med Ausbild concerning the appreciation of the journal [6] already generated success. The answers to the following questions should provide information on that:

- What are the universities the last authors publishing in the GMS Z Med Ausbild are affiliated to?
- How did the citation frequency of GMS Z Med Ausbild articles progress in comparison to articles issued in comparable open access journals that focus on medical education research in English-speaking countries?

## 2. Methods

### 2.1. Analysed publications

Among all articles published as original papers, project reports, reviews or position papers in the GMS Z Med Ausbild in 2007-2015 articles addressing scientific questions, i.e. studies and “studies in a broader sense”, were identified (“studies in a broader sense” were e. g. descriptions of projects that collected, processed and reported data conforming with scientific requirements, such as the evaluation findings in [10]). Other publications such as editorials or book reviews were not regarded.

#### 2.2. Material and Parameters for the Publication Analysis

The numbers of the submitted and published scientific articles (original papers, project reports and reviews) were selected from the GMS submission tool (Manuscript Operating System, MOPS) for the period 2007-2015 (for the definition of the types of publication see “Information for Authors” on the GMS Z Med Ausbild/J Med Educ website via the following link [http://www.egms.de/en/journals/zma/authors.htm]). Rejection rates were calculated with the statistics obtained from MOPS. Hereby a distinction was made between a “definite” rejection and a rejection with the option for resubmission as potential quality indicators of the submitted papers. Last authors and their university affiliation were encoded as stated in the respective articles. The MOPS system allows access to publications of the GMS Z Med Ausbild since 2007, therefore, data for the period of 2007-2015 was collected.

Using the Delphi Method a checklist for the analysis of manuscripts was developed covering general information (year of publication, authors and their affiliations) and information on the type and quality of the study. Among others – based on Ringsted et al. – the theoretical framework (yes/no), study type (explorative, experimental, observational study, translational study), data collection (retrospective, prospective, not visible, inapplicable), presentation of the findings (verbalised, supported by figures, still pending, none) and scope of the findings (local, German-speaking countries, international) were taken into account [11]. The checklist was then realised as an online format.

#### 2.3. Citation Frequency

The citation frequency was regarded as a parameter indicating the appreciation of an article and/or a journal. Citation frequency of articles published in GMS Z Med Ausbild during the first five years after being listed on PubMed was obtained from the Web of Science® [http://apps.webofknowledge.com]. For comparison respective data for the journal BMC Medical Education (BMC Med Educ) was analysed. 

#### 2.4. Evaluation of the Publications

Three evaluators read the abstracts and categorized the published manuscripts with the help of the checklist on an online platform. In instances where the abstracts were not conclusive regarding the criteria the full text was consulted. To determine the reliability of this data 16 articles (10% of the total of the analysed publications) were judged independently from each other by two of these evaluators.

#### 2.5. Statistical Analysis

The agreement of the evaluators regarding the publication analysis was determined by the calculation of Kappa (κ), with values of >0.4 considered acceptable [12]. Data on the quantity and frequency of publication types, study types and mode of data collection were analysed descriptively. Absolute and percentage frequencies were identified for this purpose. Mean values were compared using the Student’s t-test. Distributions were compared on the basis of contingency tables by means of the Fisher's exact test. P-values <0.05 were considered statistically significant. 

## 3. Results

### 3.1. Number of Submitted and Published Manuscripts

**Figure 1:**
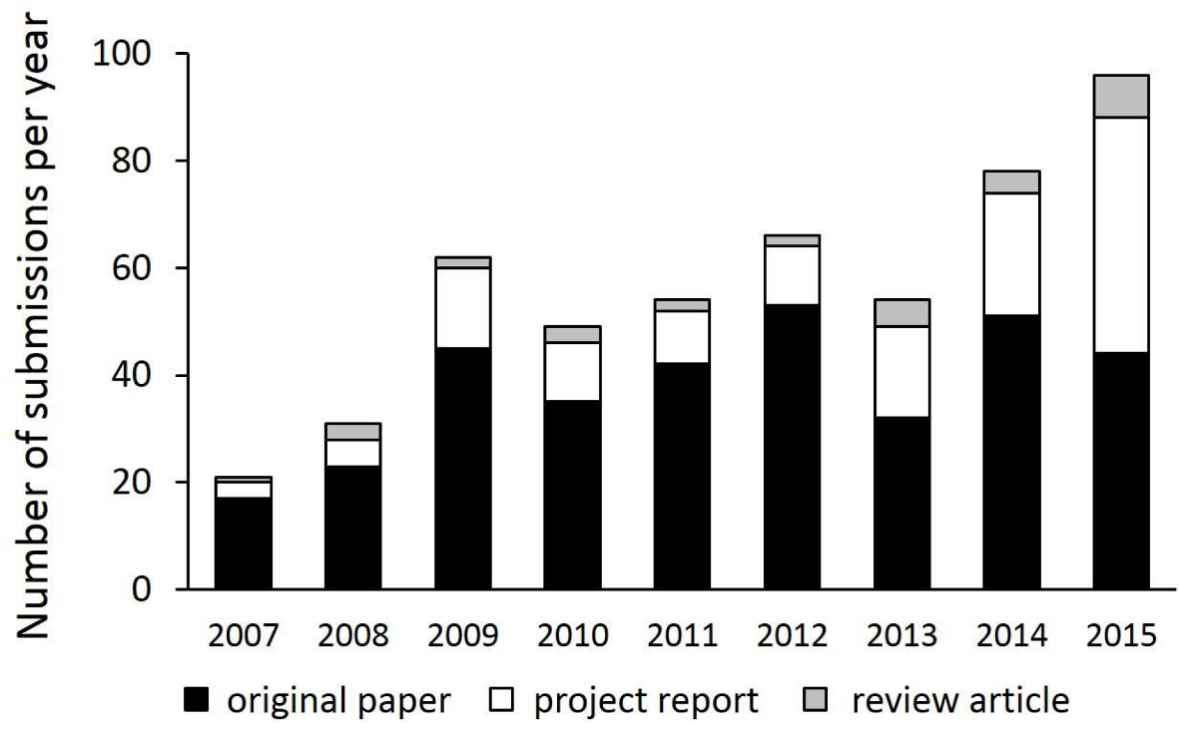
Absolute number of manuscripts submitted to GMS Z Med Ausbild in the period 2007-2015.

The number of submissions increased steadily from 2007- 2015 (see Figure 1 (Fig. 1)). During this period a total of 269 articles were published as original papers, project reports, reviews or position papers, respectively. 161 of those were deemed to be scientific submissions and thus were further analysed. Regarding the number of publications there is a striking increase in 2010. Subsequently, however, the numbers are mostly constant (2007-2009: 23.3±1.5 per year; 2010-2015: 33.8±2.8 per year; 2007-2015: 30.3±5.7). 

#### 3.2. Rejection Rates

**Figure 2:**
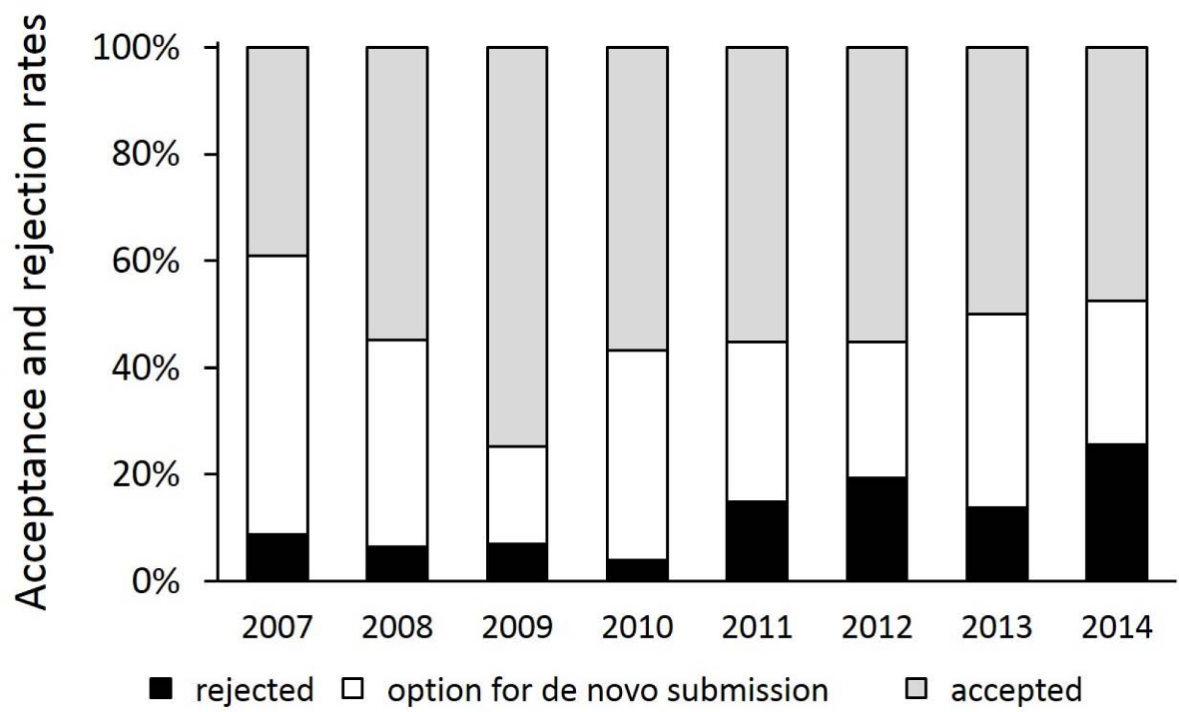
Acceptance and rejection rates of manuscripts submitted to GMS Z Med Ausbild in the years 2007 until 2014. Of note, a distinction is made between “rejected” and “rejection with the option for de novo submission”.

Only rejection rates until 2014 were considered, as there was not yet a definite decision on all of the manuscripts submitted in 2015 at the time this paper was written. Similar numbers of manuscripts submitted during 2007-2014 were rejected or accepted for publication, respectively (46±10% vs. 54±10%; see Figure 2 (Fig. 2)). There is a strikingly low rejection rate in 2009 (25%). Of note, there was an increase in rejections without the option for resubmission from 4% in 2010 (9% of all rejections) to 26% in 2014 (48% of all rejections).

#### 3.3. Frequency of Publication Types

**Figure 3:**
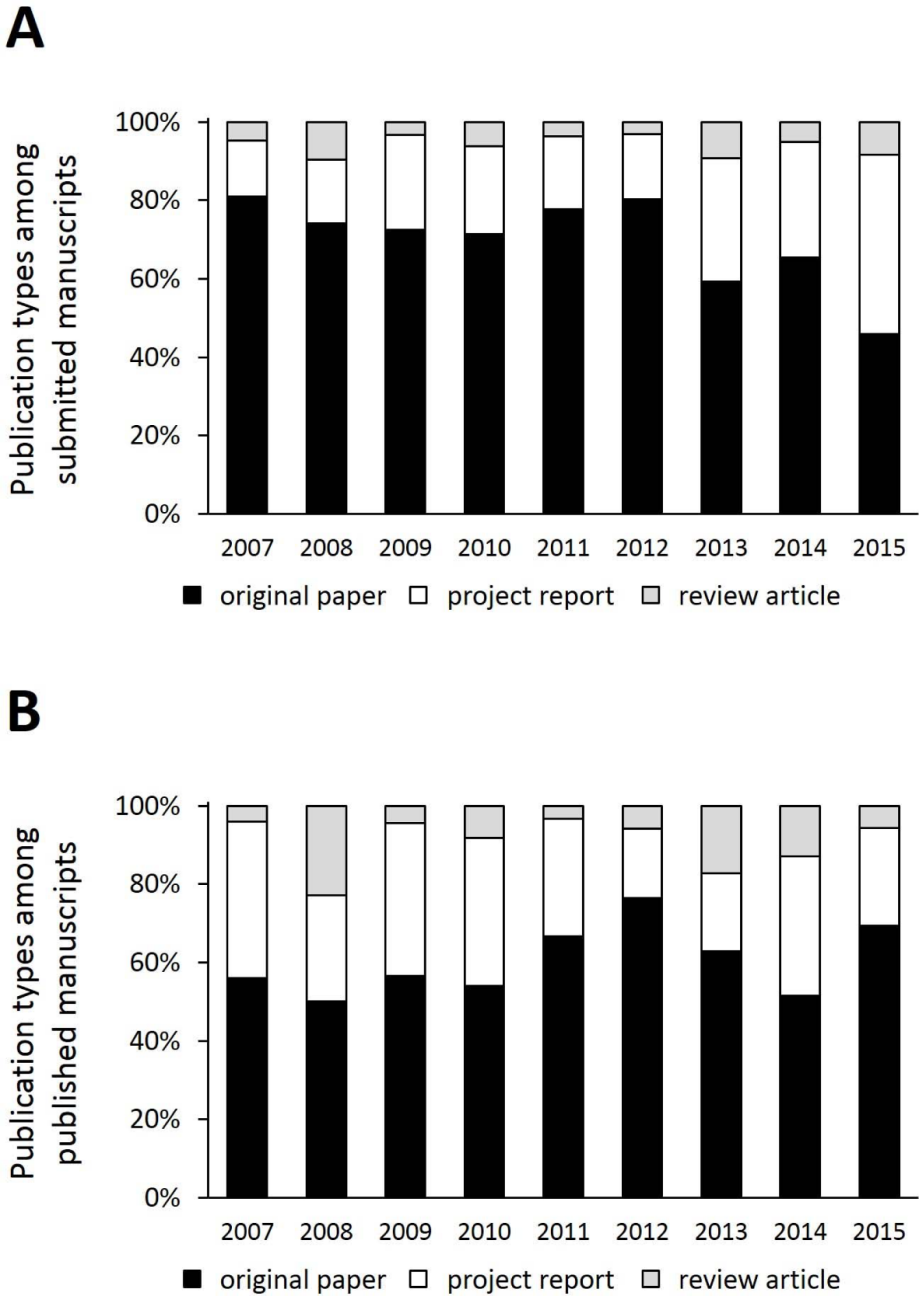
Publication types (original paper, project report, review article) in manuscripts submitted to (A) or published in (B) GMS Z Med Ausbild from 2007 to 2015.

Regarding the type of publication predominantly original papers were submitted (70±11%). The amount of project reports and review articles was 24±10% and 6±3%, respectively. It is noteworthy that there were relatively more original papers in the years 2007-2012 than there were since 2013 (76±4% and 57±10%, respectively; p<0.01; see Figure 3 (Fig. 3), Point A). In the comparison of both periods, however, the absolute number of submitted projects reports (9±4 vs. 28±14, p<0.05) and reviews (2±1 vs. 6±2, p<0.01) did increase, while the amount of submitted original papers stayed mostly the same (36±14 vs. 42±10, p=0.49). Original papers were the predominant publication type regarding actually published manuscripts, too (60±9%; see Figure 3 (Fig. 3), Point B). Here the amount of project reports and reviews was 30±8% and 9±7%, respectively. Neither rapid nor steady changes can be identified here.

#### 3.4. Study Type and Mode of Data Collection of Published Papers

**Figure 4:**
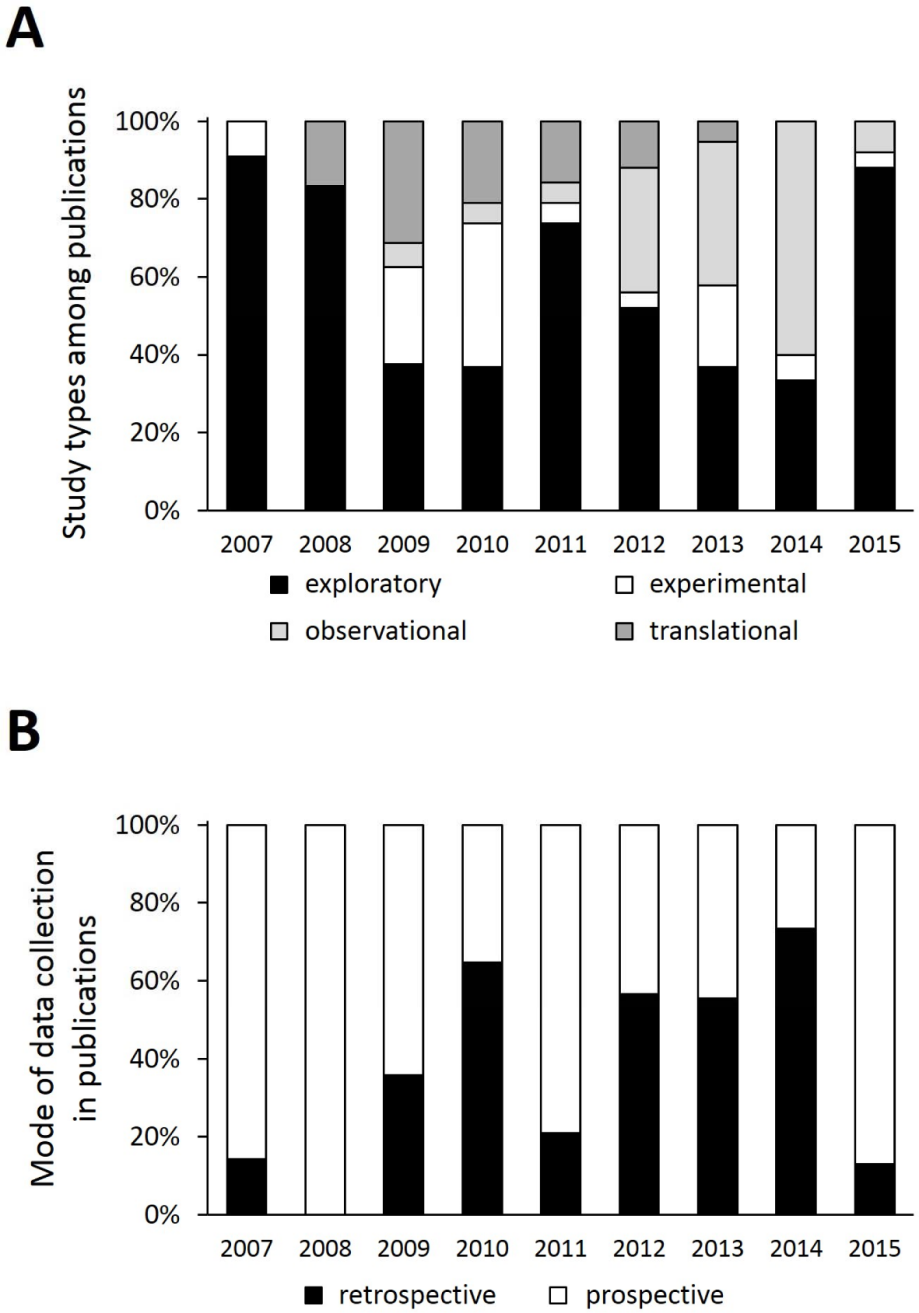
Study type (A) and mode of data collection (B) in scientific papers published in GMS Z Med Ausbild from 2007 to 2015.

Regarding evaluators’ agreement the parameters “study type” (κ=0.44) and “mode of data collection” (κ=0.67) were considered suitable for further analysis. The remaining three parameters (theoretical framework [κ=0.33], presentation of the findings [κ=0.32], scope of the findings [κ=0.03]) showed an insufficient inter-rater reliability and thus were not considered further. The amount of the various study types in the analysed publications was subject to heavy fluctuations from one year to the next (see Figure 4 (Fig. 4), Point A). Overall the most frequent study type is the explorative study (59±25%). In second place comes the observational study (17±21%), with a particularly large amount in the years 2012-2014 (43±15%). For 11% of the publications considered for our analysis it is not apparent, whether the data was collected retrospectively or prospectively or this parameter is not applicable to this publication or this type of study. The remaining publications are predominantly based on prospectively collected data (59%). However, a wide variance can be seen over the whole period (see Figure 4 (Fig. 4), Point B).

#### 3.5. University Affiliation of Last Authors 

**Figure 5:**
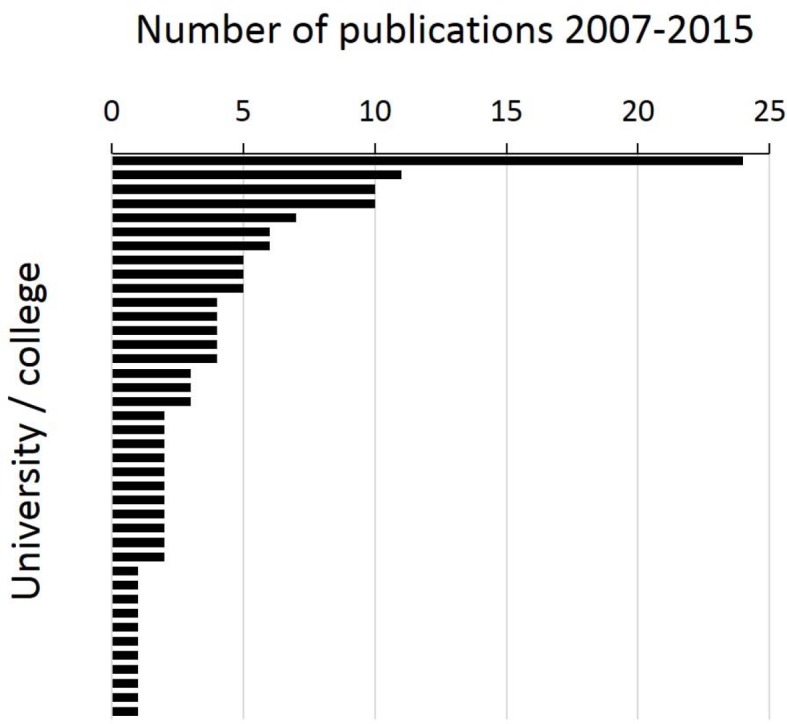
Number of scientific papers published in GMS Z Med Ausbild during 2007-2015 sorted according to last authors’ affiliations.

Since last authors usually initiate, conceptualise and supervise a study and are most likely permanently employed their affiliations were analysed to allow for a statement on the distribution of the research activity underlying publications in GMS Z Med Ausbild. This analysis shows that 90% of all publications are from Germany, 6% from Switzerland and 4% from Austria. 39 different German-speaking universities were identified as places of origin. The number of publications coming from each university, however, differs vastly (see Figure 5 (Fig. 5)). Of all published articles 16% can be traced back to one single university and 36% to the four most frequently contributing universities. During the considered period 12 universities (31%) were represented by only one last authorship. There was no difference regarding the frequency of each type of study and mode of data collection between the (based on last authors’ affiliations) four most frequently publishing and the remaining universities. The findings of an analysis of first authors correspond in most parts with those of the last authors (not shown). 

#### 3.6. Development of the Citation Frequency 

**Figure 6:**
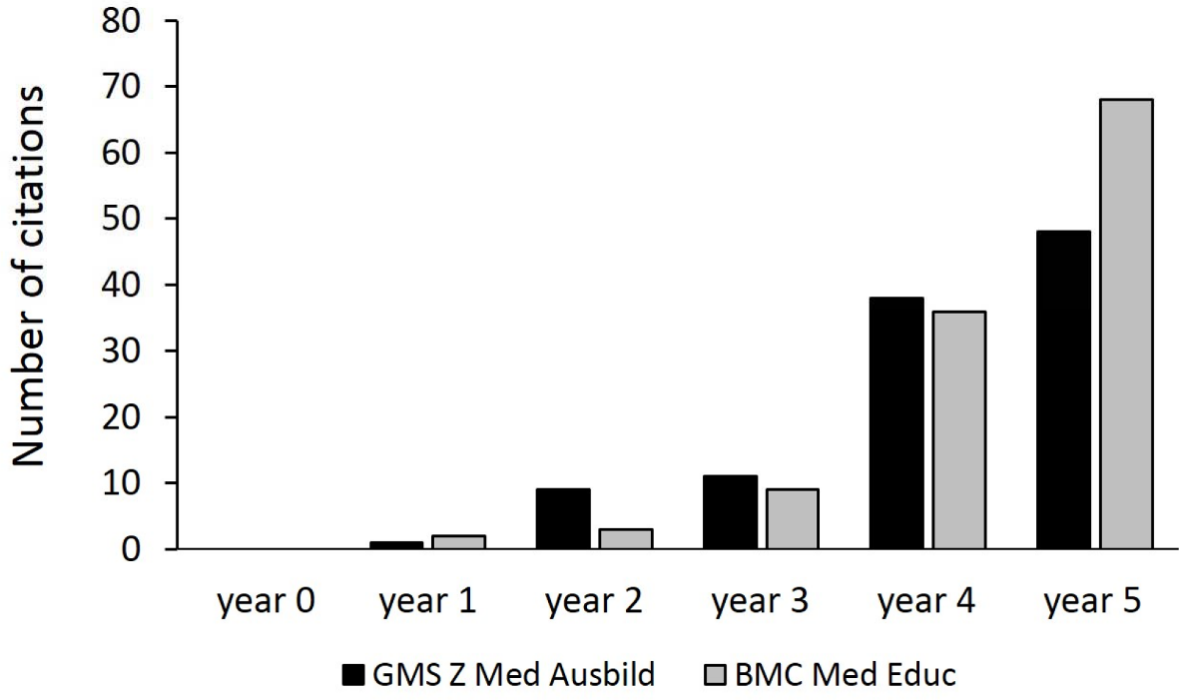
Citation frequency of articles published in GMS Z Med Ausbild (black) and BMC Med Educ (grey) in the five years after their respective PubMed listing (2011-2015 and 2002-2006, respectively).

Since the year of the PubMed listing of GMS Z Med Ausbild a steady increase in citations can be observed (see Figure 6 (Fig. 6)). The development of the number of citations in the first five years following PubMed listing was similar when comparing GMS Z Med Ausbild and BMC Med Educ.

## 4. Discussion

On the background of an internationally observable increase in significance of medical education, we analysed scientific articles submitted to and/or published in GMS Z Med Ausbild in 2007-2015 in regard to study type, publication type, last authors’ affiliation and citation rates. 

### 4.1. Submissions and Publications

Over the investigated period the number of submissions increased steadily, while the rejection rate stayed more or less the same. However, one has to keep in mind that the year of submission, the year of the final decision as well as the date of the actual publication do not always correspond. In regard to both the submitted manuscripts and the published articles original papers are the predominant type of publication, followed by projects reports and then reviews. Matching earlier observations [13] an increase of original papers among the published articles can be observed since 2007. Considering submissions since 2012 particularly the numbers of project reports and reviews are increasing, which mostly explains the decline of the relative amount of submitted original papers since then. The increasing number of submissions in the categories “project report” and “review” can be seen as a sign of the growing significance of medical education research: A great(er) number of people who focus on medical education, think it reasonable to present their experiences systematically. Regarding the type of publication a comparison of the GMS Z Med Ausbild with other journals on medical education research is in our opinion not purposive given the varying requirements and options. The distribution of the published papers regarding the various study types fluctuates significantly from one year to the other. In general, however, the explorative study is predominant. The high number of explorative studies might be explained by the German-speaking education research still being at a rather early stage in international comparison. However, in international English-speaking journals on medical education research 26% of publications from German-speaking countries are experimental studies, a value almost twice as high as in the GMS Z Med Ausbild [2]. It is tempting to speculate that authors prefer to submit high quality articles to journals listed in the Journal Citation Reports® (i.e. with an impact factor).

#### 4.2. Citation Frequency

The activities of the GMS Z Med Ausbild regarding their (national as well as international) visibility seem to show the first signs of success: within the first five years after being listed in PubMed the citation frequency of papers published in the GMS Z Med Ausbild increased steadily. This increase was similar to that of BMC Medical Education during a comparable period. Just like the GMS Z Med Ausbild this journal has not been listed in PubMed that long and it is an open access journal, too. It should be noted that the citation frequency depends on a multitude of further factors, such as the number of published articles in a certain time frame. 

#### 4.3. University Affiliation of the Last Authors

It is striking that (in regard to the last author) only a few universities are responsible for the majority of publications in the GMS Z Med Ausbild. This raises the question why other universities publish in this journal less or not at all. There are two evident hypotheses:

1. Authors from other universities prefer other journals for their publications. However, Ackel-Eisnach et al. obtained results similar to ours when analysing publications in international English-speaking journals regarding first and last authors [2]: in their and our analyses three universities rank among the five most frequently publishing universities. It cannot be ruled out that articles were alternatively published in not primarily medical education research oriented or in non-medical (e. g. education science oriented or psychological) journals.
2. The more frequently represented universities produce more and/or higher quality manuscripts overall. In a not a priori planned analysis of subgroups for the parameters “study type” and “mode of data collection”, however, no difference between the GMS Z Med Ausbild publications from the four most represented and the other universities can be observed.

To gain authors that are not from Germany, Austria or Switzerland is an important aspect of the international competitiveness of the GMS Z Med Ausbild/J Med Educ. At least regarding the last authorship in this respect no effect was (yet) observable during the period examined. Being included in the Journal Citation Reports® and thus receiving an impact factor would be an important step regarding the appeal of GMS Z Med Ausbild/J Med Educ and stays a proclaimed goal of the publisher [8]. Nevertheless, the citation frequency itself can be incentive enough for (potential) authors to (further) publish in the GMS Z Med Ausbild/J Med Educ. The Hirsch index (h-index), for example, as another popular parameter for the evaluation of scientific relevance, relates to the citation frequency and the number of an author’s publications and is calculated independently from the impact factor [14], [15], [16]. Of note, both the impact factor and the Hirsch index are based on the number of citations. Actually, publications are perceived considerably more often. In the case of the GMS Z Med Ausbild for example, this can be taken from the (steadily increasing) numbers of online access in the years 2010-1014 [7].

#### 4.4. Limitations of the Research

Three of the five parameters that were initially intended for evaluation of the published articles could not be assessed reliably enough and thus were not regarded further. Reasons for that could have been unclear item definitions or an insufficient agreement of the evaluators in advance (“evaluator training”). Hence, interesting aspects regarding expenditure, quality and significance of education research remain open. It is also worthwhile noting, that data from only two evaluators were the basis for the calculation of evaluator agreement. A further limitation of this research is that the significance of medical education research is not only represented by the type of the published studies, but also by a number of other criteria, that were not analysed (e.g. topic of research, research question, transferability of the findings) [11]. The quality of the published research findings cannot be judged definitely on the basis of the parameters that we investigated, e.g. as not the applied methodology itself but its adequacy is crucial. Qualitative interviews in the faculties may offer a more detailed and structurally deeper insight regarding the significance and the quality of medical education research. It is to be expected that further ideas for relevant criteria can be generated from such interviews. Another limitation is that the reception and the significance of the GMS Z Med Ausbild was evaluated on the basis of the criteria “citation frequency” and “university affiliation of the author” which is only a small selection of potentially relevant indicators.

## 5. Conclusions

The growing number of manuscripts on education research submitted to the GMS Z Med Ausbild shows that people from German-speaking countries who are involved in medical education increasingly invest time and resources to contribute to the scientific discourse of teaching and learning in medicine. The visibility of the journal rises as shown by the citation frequency and number of online access. Taking this into account GMS Z Med Ausbild/J Med Educ as a journal for medical education and education research with a focus on topics concerning German-speaking countries can be considered as well established. Important steps for the international competitiveness have been taken, including the PubMed listing, the (in the meantime renewed) application for the allocation of an impact factor as well as the improved evaluation process [8], [13], [17]. The analysis of various bibliometric indicators beyond the impact factor, intended to compare the current position of GMS Z Med Ausbild/J Med Educ and other international journals in the area of medical education, is highly anticipated [8].

## Competing interests

The authors declare that they have no competing interests.
